# Traité International de Psychologie pathologique

**Published:** 1912-12

**Authors:** 


**Traité International de Psychologie pathologique.** Directeur : Dr. A. Marie. Tome iii. Pp. viii, 1086. Paris : Félix Alcan. 1912. Price 25 fr.

This the third and last volume of the series opens with a chapter by Professor Bianchi dealing in general considerations of the physio-pathology of the sensorial sphere ; the origin and evolution of sensation, perception, emotion and intelligence ; association processes ; morbid perversions of all and each of these ; site and construction of hallucinations, etc. Siborski deals with the objective study of normal and morbid intellectual, emotional and volitional states ; racial characteristics, changes in organic functions, orders and disorders of speech and allied mechanisms ; studies of subconcious states, of the ego ; the relations between psychic and somatic conditions. There is detailed observation of physiognomy, gestures, pulse and respiration changes ; attention and fatigue, association and disassociation and their correlative physical changes. Numerous illustrations drawn from celebrated pictures or sculpture exemplify the remarks on the physical expression of mental states. In succeeding chapters are considered the psychopathies of history, the psychoses of different countries and varieties due to special conditions are compared; collective or epidemic insanities, the psychology and morbid psychoses of animals, are studied also from comparative standpoint.

There is a very complete survey of the etiology of insanity, setting forth meteorological, social and particular physical influences in most elaborate detail. The final chapter is devoted to a description of laboratory methods of investigation.

This volume, like its predecessors, is exhaustively complete, and somewhat discursive. It is essentially a work of reference, and a reliable one. It epitomises all recognised continental views on the subjects treated. There is about the same recognition of English work as there is in English text-books, and one cannot, therefore, comment on any prejudice shown.

